# Comparison of “Continuous” and “End-ischemic” Approaches of Oxygenated Hypothermic Machine Perfusion for Pancreas Preservation

**DOI:** 10.1097/TXD.0000000000001888

**Published:** 2026-01-15

**Authors:** Mohamed Aly Mohamed Elshafei Elzawahry, John Fallon, Benoit Hastoy, Maria Letizia Lo Faro, Sameena Nawaz, Julien Branchereau, Anne Clark, Peter Friend, Rutger Ploeg, James Hunter

**Affiliations:** 1 Nuffield Department of Surgical Sciences, University of Oxford, Oxford, United Kingdom.; 2 Oxford Centre for Diabetes, Endocrinology and Metabolism (OCDEM), University of Oxford, Oxford, United Kingdom.; 3 Institut de Transplantation Urologie Néphrologie (ITUN), CHU Nantes, Nantes, France.; 4 Centre de Recherche en Transplantation et Immunologie UMR 1064, INSERM, Université de Nantes, Nantes, France.; 5 Nuffield Department of Surgical Sciences, Oxford Biomedical Research Centre, University of Oxford, Oxford, United Kingdom.

## Abstract

**Background.:**

Pancreas transplantation can successfully restore physiological insulin production, but it comes with a potentially significant morbidity burden. Pancreas grafts are extremely vulnerable to ischemia/reperfusion injury. Investigating technologies that aim to mitigate ischemia/reperfusion injury, such as oxygenated hypothermic machine perfusion (HMPO_2_) for pancreas preservation and their modes of application, is crucial to inform clinical translation.

**Methods.:**

A circulatory death porcine model was used to compare different HMPO_2_ modes for pancreas preservation. Eighteen porcine pancreases were allocated to 3 experimental groups: static cold storage (SCS), continuous HMPO_2_, and end-ischemic HMPO_2_. Normothermic reperfusion (NR) was used for assessment.

**Results.:**

The ischemic times and wet:dry ratios were comparable among the 3 groups. Perfusate flow increased throughout NR and was highest at the end of NR across all groups, with no significant difference between the groups. Amylase, lipase, lactate dehydrogenase, and cell-free DNA showed no significant differences in the pattern of change among the groups. Red blood cells were present consistently in vessels and islet capillaries at the end of NR in all pancreases. The continuous HMPO_2_ group showed significantly higher biphasic insulin secretion in response to glucose stimulation (*P* = 0.01).

**Conclusions.:**

Continuous HMPO_2_ is a superior method for preserving beta-cell function compared with SCS and end-ischemic HMPO_2_ in a porcine model of circulatory death when assessed by NR with whole blood as a surrogate for transplantation. As end-ischemic HMPO_2_ is also feasible and potentially superior to SCS, testing its safety would be an excellent first step in translating this technology into clinical practice.

## Graphical abstract

**Figure:**
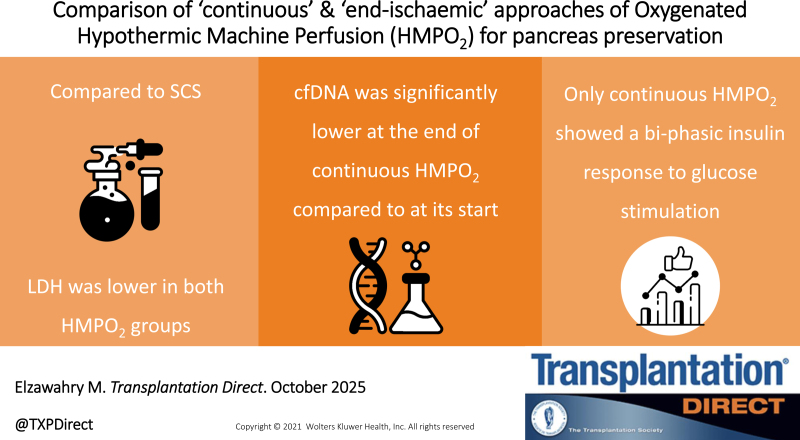

## INTRODUCTION

### Diabetes Mellitus and Pancreas Transplantation

Diabetes mellitus significantly reduces both quality of life and life expectancy. The current standard of care does not prevent long-term complications in many patients, including nephropathy, neuropathy, limb loss, and cardiovascular disease.^1^ The occurrence and progression of long-term secondary complications of diabetes correlate with the quality of blood glucose control.^2^ Pancreas transplantation is the only method approved for clinical practice, which can consistently reestablish physiological insulin production.^3^

Despite substantial improvements in pancreas graft and recipient outcomes, the proportion of pancreases retrieved that result in a transplant, termed “utilization,” is the lowest among solid abdominal organs. In the United Kingdom, >50% of pancreases retrieved for the purpose of transplantation are not implanted.^4^ The reason for this is primarily clinician uncertainty about organ quality and concern over potential postoperative complications, namely graft pancreatitis.

### Pancreas Vulnerability During Preservation

The pancreas is extremely vulnerable to ischemia/reperfusion injury (IRI), which is a key factor in the pathophysiology of early posttransplant morbidity and mortality, particularly graft pancreatitis. This has important clinical implications, such as reoperation after pancreas transplant, which occurs in about 20%–25% of patients, and pancreatitis remains 1 of the main causes of early graft loss.^5,6^

The current standard for storage and transport of donor pancreas grafts is static cold storage (SCS). During SCS, ATP is depleted, and the toxic byproducts of anaerobic respiration, such as succinate and lactate, accumulate in the tissue. When the pancreas is transplanted and reperfused with recipient blood, IRI occurs, accompanied by free radical production, and the severity of this cold ischemic injury is unmasked.^7^

### Oxygenated Hypothermic Machine Perfusion for Organ Preservation

Oxygenated hypothermic machine perfusion (HMPO_2_) has been successfully used to mitigate the effects of IRI in clinical liver^8^ and kidney^9^ transplantation. HMPO_2_ delivers oxygenated perfusion solution through the organ under hypothermic conditions (<12 °C). This combines the safety of hypothermia with the benefits of dynamic machine perfusion, delivering oxygen to support mitochondrial function, reducing IRI by flushing out toxic metabolic byproducts, and avoiding the accumulation of mitochondrial succinate and subsequent production of reactive oxygen species.^7^

Clinically, HMPO_2_ for the pancreas is due to be assessed in a safety and feasibility clinical trial, which is yet to start recruitment.^10^ It has been investigated in various preclinical models of porcine, nonhuman primate, and human pancreas grafts and assessed using multiple perfusion parameters, perfusate composition, and methods of oxygenation, showing feasibility and potential benefit.^11-17^ There is no consensus on the mode required for improved pancreas preservation, whether from the start of hypothermic preservation, the continuous mode, which has shown benefit in kidney preservation^9,18^ or if it is sufficient to oxygenate the pancreas for a period after a small number of hours in SCS before transplantation, the end-ischemic mode, which has shown benefit in liver preservation.^8^

### Aim and Research Questions

Our aim was to assess and compare different modes of preservation for porcine pancreas grafts obtained after circulatory death using HMPO_2_. Specifically, to answer the following questions:

1. Is HMPO_2_ superior to SCS in the preservation of pancreases?
2. Is there a benefit from starting the HMPO_2_ of the pancreas at retrieval (continuous HMPO_2_) versus after transport in SCS to the implant site (end-ischemic HMPO_2_)?

## MATERIALS AND METHODS

Porcine pancreases (n = 18) were retrieved from domestic pigs with a weight between 45 and 72 kg at a UK abattoir. Animal death was complied with the Welfare of Animals at the Time of Killing (England) Regulations 2015 and the retained EU Regulation 1099/2009; therefore, no further ethical approval or license was required to perform this research.

### Procurement and Surgical Preparation

After regulated stunning and exsanguination, 1 L of whole blood was collected and anticoagulated with 10 000 IU of unfractionated heparin sodium (1000 units per 1 mL, Wockhardt UK Ltd). The surgical technique of pancreas retrieval previously published by our group was performed, using 1 L of University of Wisconsin Cold Storage Solution (Carnamedica, Poland).^14,19^ Refinements to the retrieval technique included (1) cannulating the superior mesenteric artery and celiac axis, instead of an aortic tube, using 10 Fr cannulas for organ perfusion (Carnamedica, Poland) and (2) not using a stapling device but applying a vascular clamp to the root of mesentery and controlling it with a running 0 PDS *Plus* (Johnson & Johnson Medical N.V., Belgium) suture.

After surgical preparation, 6 pancreases were allocated to each of the 3 experimental groups for 8 h of hypothermic preservation:

1. Control group: SCS for the totality of the hypothermic preservation time.
2. Continuous group: HMPO_2_ for the totality of the hypothermic preservation time.
3. End-ischemic group: SCS followed by HMPO_2_ for the last 2 h of the hypothermic preservation time.

**FIGURE 1.**
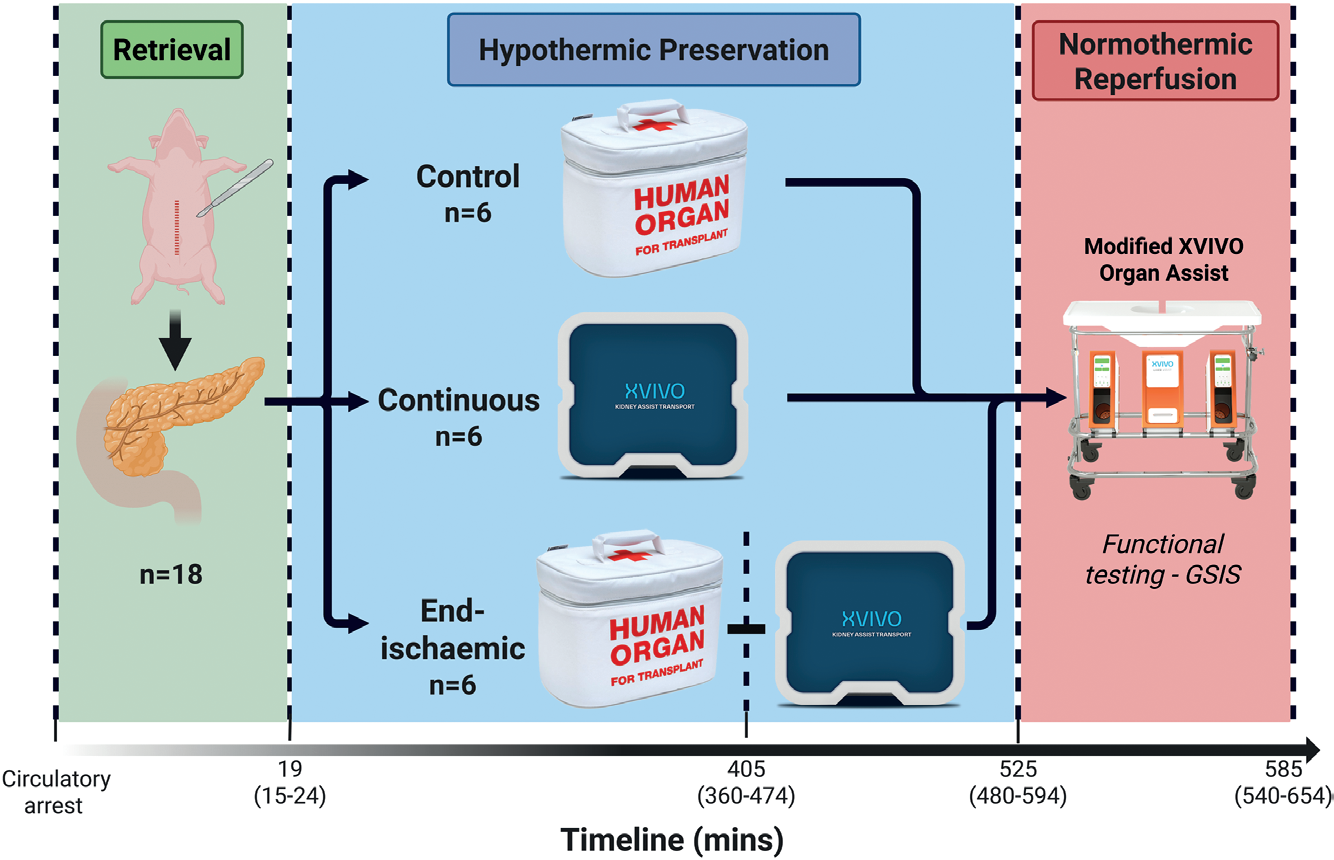
Flowchart of experimental design. The 3 groups in the study are depicted with the duration of each preservation period. The control (SCS) group was preserved in SCS for the entire hypothermic preservation time. Continuous HMPO_2_ group was preserved using HMPO_2_ for the entire hypothermic preservation period. End-ischemic HMPO_2_ group was preserved using SCS, then HMPO_2_ for the last 2 h of hypothermic preservation. Each group then underwent 60 min of normothermic reperfusion, including functional testing with glucose-stimulated insulin secretion (GSIS). HMPO_2_, oxygenated hypothermic machine perfusion; SCS, static cold storage.

After hypothermic preservation, all 18 pancreases underwent normothermic reperfusion (NR) with autologous whole blood for 60 min (Figure 1).

### Oxygenated Hypothermic Machine Perfusion

Pressure-controlled pulsatile perfusion was delivered via the superior mesenteric artery and celiac axis cannulas at a set systolic pressure of 25 mm Hg with a temperature range of 4–8 °C on a modified XVIVO Kidney Assist Transporter device using University of Wisconsin Machine Perfusion Solution (Carnamedica, Poland). This device was selected to accommodate modifications designed to suit the pancreas and to optimally deliver oxygen. These include an in-house 3D-printed organ chamber and a polymethylpentene membrane oxygenator (MEDOS HILITE 2800 LT) with oxygen delivered via a medical-grade oxygen cylinder (BOC, Linde Group, Surrey, United Kingdom) at a flow rate of 0.5 L/min. This resulted in a perfusate partial pressure of oxygen (pO_2_) ≥150 mm Hg (~20 kPa). The portal vein drained passively into the reservoir at the bottom of the organ chamber, and the perfusate was then recirculated.

### Normothermic Reperfusion

As a surrogate for transplantation, we used autologous heparinized whole blood to perfuse pancreases after the hypothermic preservation phase. NR was set up using a modified XVIVO Kidney Assist device, based on our previous work.^14^ The modifications included using the same 3D-printed organ chamber used for HMPO_2_, adding a bypass, and a flow aim of 100 mL/min with a maximum pressure setting of 60 mm Hg for 1 h.

The NR circuit was primed with 900 mL of heparinized whole blood with verapamil and 0.9% saline to achieve a hematocrit of 0.35. Oxygenation with 21% oxygen, 5% carbon dioxide, and 74% nitrogen at a flow rate of 0.5 L/min was delivered to the polymethylpentene membrane oxygenator (MEDOS HILITE 2800 LT), which is integrated with a bubble trap to enable gas exchange and provides the surface area to keep the perfusate at 37 °C via connection to the heat exchanger unit of the device.

### Sample Collection and Analyses

#### Perfusate

Perfusate samples were collected at baseline, at the end of hypothermic preservation, and end of NR. During NR, the collected perfusate samples were centrifuged at 1800*g*, to separate the red blood cell (RBC) component, and the supernatant was sampled. Perfusate samples were stored at –80 °C, then thawed and analyzed as a batch to investigate markers of tissue damage. Amylase, lipase, and lactate dehydrogenase (LDH) were analyzed in the ARCHITECT c Systems (Abbott GmbH & Co. KG, Germany) using the following inserts: Amylase2 (Abbott Ireland, Ireland), lactate dehydrogenase2 (Abbott Ireland, Ireland), and lipase (Abbott GmbH & Co. KG, Germany). Circulating cell-free DNA (cfDNA) was isolated using the QIAamp Circulating Nucleic Acid kit (Qiagen, Venlo, Limburg, Netherlands) following manufacturer’s instructions. The isolated cfDNA was then quantified by the Circulating DNA Quantification Kit (Abcam Ltd, Cambridge, United Kingdom) following manufacturer’s instructions.

#### Biopsies

Wedge tissue samples were removed at baseline, at the end of hypothermic preservation, and at the end of NR. Tissue samples were used fresh to determine wet:dry weight ratio, which is a measure of tissue fluid content to indirectly assess parenchymal edema, as described in.^14^ Wedge tissue samples for histology were removed at the same timepoints, fixed in 10% formal saline, and stored at 4 °C before wax embedding. 5-µm sections were stained using hematoxylin and eosin to assess histological appearance, tissue architecture, and the presence of RBCs in vasculature by 2 blinded assessors. Insulin retention in islet beta cells was determined by immunolabeling for insulin using an “in-house” anti-insulin antibody raised in a guinea pig, fully cross-reactive with porcine insulin, previously validated and used by our group^20,21^ and visualized with peroxidase/3,3′-diaminobenzidine. To characterize other islet hormones, sections were simultaneously immunolabeled for insulin (antibody as above), glucagon (mouse anti-glucagon, SIGMA), and somatostatin (rabbit anti-somatostatin, DAKO, Agilent, United Kingdom) and fluorescently-labeled secondary antibodies: anti-guinea pig 633, anti-mouse 568, and anti-rabbit 633 (Invitrogen, MA). These labeled slides were examined with a Radiance 2000 confocal microscope (Bio-Rad, CA) to illustrate porcine islet structure.

#### Glucose-stimulated Insulin Secretion

Perfusate samples were taken, processed, and stored as described above. A 20 mM glucose bolus was administered on the arterial side of the circuit 30 min after the start of NR. Baseline samples were collected at 5 min, 3 min, and immediately before administering the bolus. Samples were then taken at 1-min intervals for the first 5 min after bolus administration, then at 5-min intervals for the following 15 min. The venous effluent was collected and sampled or discarded for the 20 min after bolus administration, and therefore not recirculated. Insulin secretion was measured using ELISA for human insulin (Mercodia, Uppsala, Sweden; n = 3 in each experimental group), following manufacturer’s instructions. Glucose was measured using a Bayer Contour Next device (Bayer, Germany).

### Statistical Analysis Plan

Data are expressed as mean ± SEM unless otherwise specified. Mixed-effects analysis with a Geisser-Greenhouse correction was used to determine differences between the groups for reporting, amylase, lipase, and lactate dehydrogenase to handle a maximum of 2 missing values per outcome. One-way ANOVA was used to determine the difference between wet:dry ratios normalized to baseline of each experiment. Mixed-effects analysis with the Geisser-Greenhouse correction was used to compare perfusate flow during NR. Two-way ANOVA with a Geisser-Greenhouse correction and a post hoc Tukey test was used to determine differences between the groups for cfDNA values normalized to the baseline of each experiment and for insulin secretion values during glucose-stimulated insulin secretion (GSIS) normalized to the mean of the 3 basal measurements (minutes –5, –3, and 0) for each experiment. All statistical tests were performed using Prism version 10.2.1 (GraphPad, CA).

## RESULTS

### Perfusion Parameters

Median warm ischemia time (WIT), defined as the time between the start of exsanguination and the time of cold perfusion with University of Wisconsin Cold Storage Solution, was 19 min in all 3 groups. WIT ranged between 15 and 22 min in the continuous group, 18 and 20 min in the end-ischemic group, and 15 and 24 min in the SCS group. The median (range) hypothermic preservation time was 493 (480–532) min in the continuous group, 532.5 (505–570) min in the end-ischemic group, and 543 (490–594) min in the SCS group (Kruskal-Wallis, *P* = 0.09; illustrated in Table 1).

**TABLE 1.** WIT and HP times

Experimental group	WIT	HP
Continuous HMPO_2_ (n = 6)	19 (15–22)	493 (480–532)
End-ischemic HMPO_2_ (n = 6)	19 (18–20)	532.5 (505–570)
Control (SCS) (n = 6)	19 (15–24)	543 (490–594)

WIT during retrieval and HP in the 3 experimental groups. Illustrated as median and range in minutes.

HP, hypothermic preservation; SCS, static cold storage; WIT, warm ischemia time.

**FIGURE 2.**
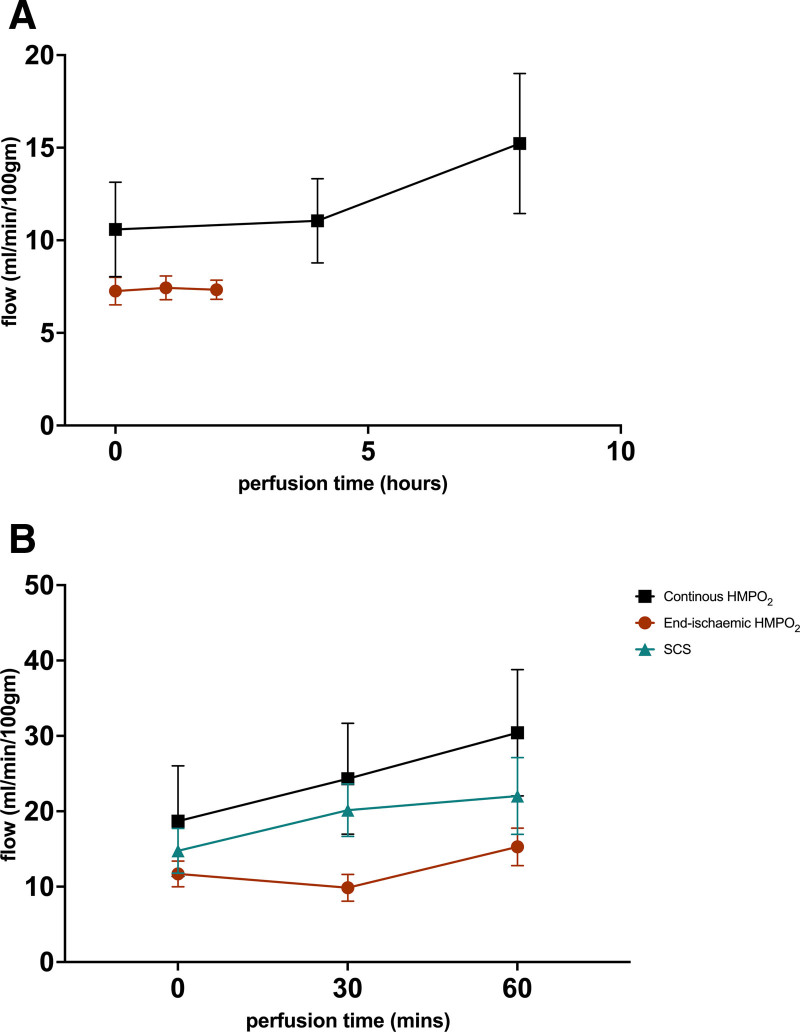
Arterial perfusate flow. A, Arterial perfusate flow (mL/min/100 g of graft weight) during continuous HMPO_2_ and end-ischemic HMPO_2_. B, Arterial perfusate flow (mL/min/100 g of graft weight) at the start, midpoint, and endpoint of NR. Values displayed as mean ± SEM. There was a significant increase in flow during NR in all 3 groups over time (mixed-effects analysis, *P* < 0.01), with no significant difference between groups. HMPO_2_, oxygenated hypothermic machine perfusion; NR, normothermic reperfusion; SCS, static cold storage.

During continuous HMPO_2_ arterial perfusate flow gradually increased through perfusion from a mean of 10.59 mL/min/100 g of graft weight to a mean of 15.23 mL/min/100 g of graft weight (Figure 2A), but not during the shorter HMPO_2_ in the end-ischemic group which started with a mean of 7.26 mL/min/100 g of graft weight and ended with a mean of 7.33 mL/min/100 g of graft weight. The perfusate flow at the end of NR across all groups was higher compared with the start of NR (mixed-effects analysis, *P* < 0.01), but there was no significant difference between the groups (*P* = 0.19; Figure 2B).

### Markers of Edema and Damage

**FIGURE 3.**
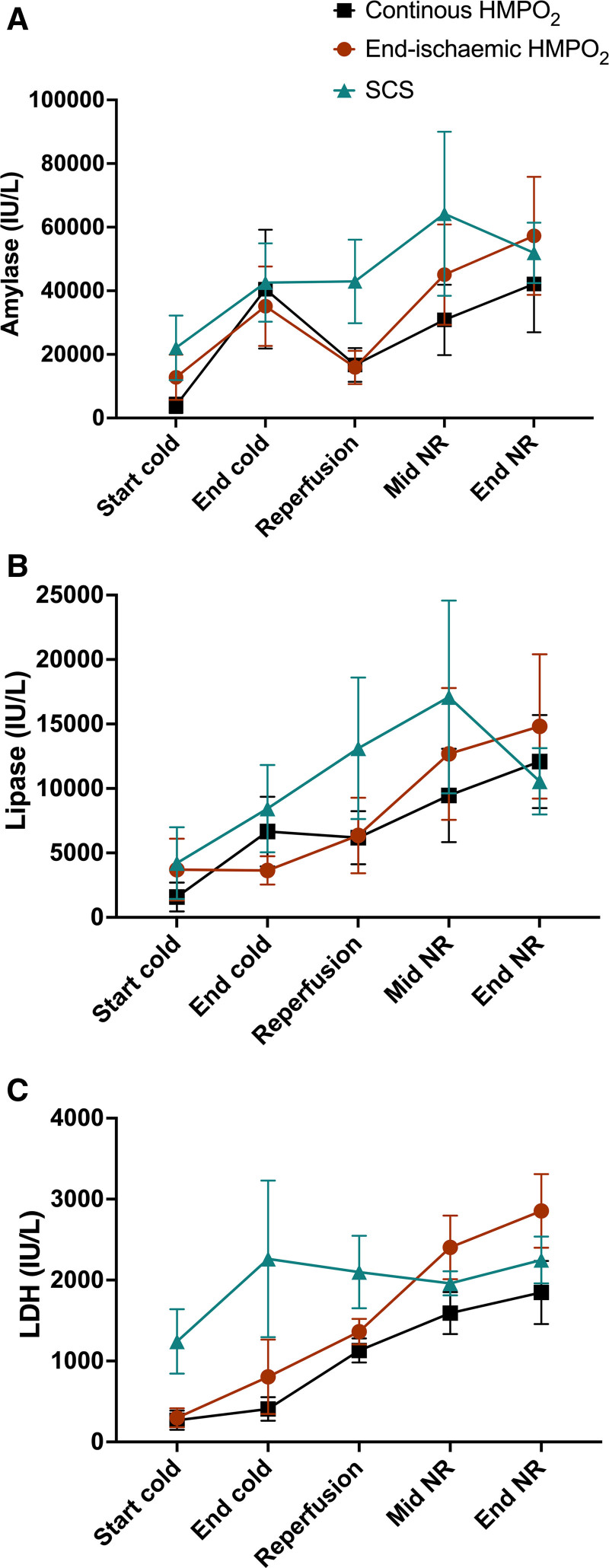
Analysis of biochemical injury markers, amylase (A), lipase (B), and LDH (C), in each group, measured at the start of cold preservation, end of cold preservation, then at start, midpoint, and end of NR. The 3 markers increased during perfusion, and there was a statistically significant difference between the 3 groups for LDH but not for amylase or lipase (mixed-effects analysis with a Geisser-Greenhouse correction, *P* = 0.03, *P* = 0.47, and *P* = 0.7, respectively). All values are displayed as mean ± SEM. “Start cold” is the start of cold preservation and “end cold” is the end of cold preservation. HMPO_2_, oxygenated hypothermic machine perfusion; LDH, lactate dehydrogenase; NR, normothermic reperfusion; SCS, static cold storage.

Amylase and lipase were not significantly different between the groups (mixed-effects analysis, *P* = 0.47 and *P* = 0.7, respectively; Figure 3A and B). All pancreases showed a statistically significant increase in these markers by the end of NR compared with baseline (mixed-effects analysis, *P* = 0.0011, 0.0017, and 0.0006, respectively), peaking at mid NR in the SCS group (Figure 3A and B). Lactate dehydrogenase (LDH) showed significantly different overall levels between the groups (mixed-effects analysis, *P* = 0.03) and change over time (*P* = 0.0006), but no significant differences were noted in the pattern of change, that is, increase, among the 3 groups (*P* = 0.13; Figure 3C).

**FIGURE 4.**
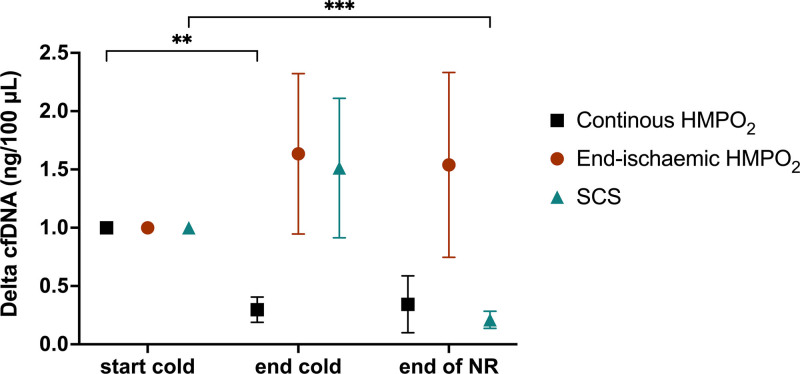
cfDNA levels in perfusate samples. cfDNA levels in each group measured at the start of cold preservation, end of cold preservation, and then end of NR. The change (delta) in cfDNA is presented as mean ± SEM for each group and has been normalized to the baseline value of each pancreas. *Levels of cfDNA were significantly different among individual pancreases (2-way ANOVA, *P* = 0.008). Multiple comparisons with post hoc correction by the Tukey test demonstrated a statistically significant decrease between start and end of cold preservation in the continuous HMPO_2_ group (**P = 0.006) and a statistically significant decrease between start of cold preservation and end of NR in the SCS group (***P = 0.0003). cfDNA, cell-free DNA; HMPO_2_, oxygenated hypothermic machine perfusion; NR, normothermic reperfusion; SCS, static cold storage.

The levels of circulating cfDNA (Figure 4) were significantly different between individual pancreases (2-way ANOVA, *P* = 0.008), but no significance was noted in the mean differences between groups, over time, or in the pattern of change between the 3 groups (2-way ANOVA, *P* = 0.24, 0.27, and 0.08, respectively). On multiple comparisons, there was a statistically significant decrease between start and end of cold preservation in the continuous HMPO_2_ group (Tukey’s post hoc, *P* = 0.006) and start of cold preservation and end of NR in the SCS group (Tukey’s post hoc, *P* = 0.0003).

There were no significant differences in parenchymal edema between the 3 groups as assessed by tissue wet:dry ratio (1-way ANOVA, *P* = 0.09). All groups demonstrated a gradual increase in parenchymal edema during the 8 h of hypothermic preservation and 60 min of NR.

### Histological Assessment

**FIGURE 5.**
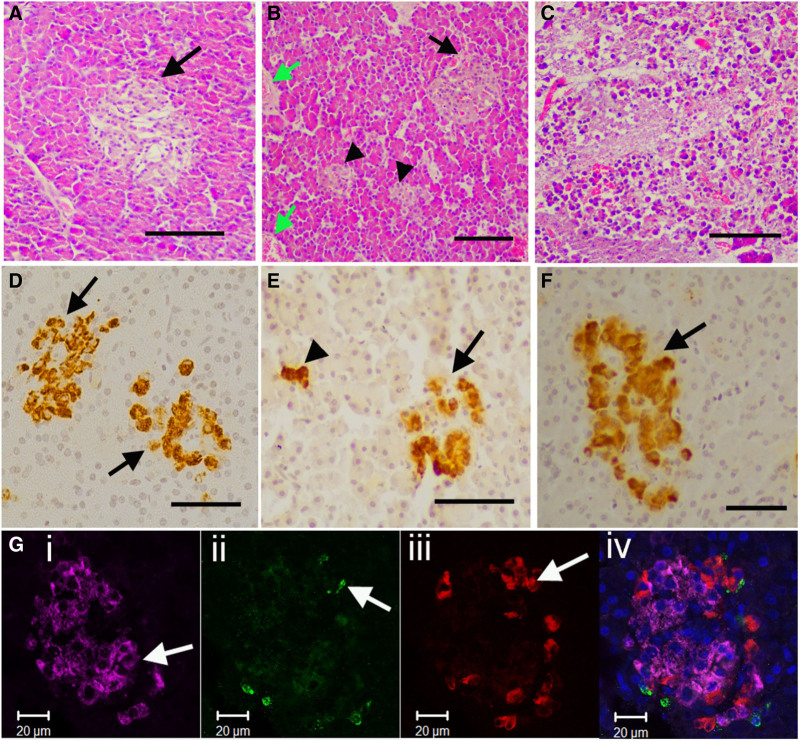
Histological assessment of pancreas wedge biopsies. A, Representative hematoxylin and eosin (H&E) staining of pancreas at the start of hypothermic preservation. Islet (arrow) does not contain any red blood cells. Scale bar 50 μm. B, Representative Pancreas from the continuous HMPO_2_ group at the end of NR. Red blood cells in islet capillaries (black arrow) and in larger blood vessels (green arrows). Small endocrine clusters (arrowheads) were typical of porcine pancreas. Scale bar 50 μm. C. Representative pancreas from the SCS group at the end of NR showing severe autolysis and exocrine damage. Scale bar 100 μm. D–F, Islets immunolabeled for insulin from pancreases in continuous, end-ischemic HMPO_2_ and SCS groups, respectively. Arrowhead in (E) shows a small cluster of endocrine cells; these contained only beta cells. Scale bars 25 μm. G, Fluorescent immunolabeling for (i) insulin: violet (arrow); (ii) somatostatin: green (arrow); (iii) glucagon: red (arrow) using confocal microscopy; and (iv) merged images with nuclear DAPI labeling. Scale bars 20 μm. DAPI, 4',6-diamidino-2-phenylindole; HMPO_2_, oxygenated hypothermic machine perfusion; NR, normothermic reperfusion; SCS, static cold storage.

Histological assessment of hematoxylin and eosin–stained sections from all pancreases by 2 blinded assessors (Table 2) showed no RBCs in vessels or capillaries at the start of hypothermic preservation in all pancreases (Figure 5A). RBCs were present consistently in vessels and islet capillaries at the end of NR in all 3 groups (Figure 5B). All pancreases showed autolysis and exocrine damage at the end of NR, which was graded as more severe in the SCS group (Figure 5C).

**TABLE 2.** Histological assessment

Experimental group	Timepoint	RBCs in capillaries	RBCs in arterioles/venules	Necrosis	Architecture
Continuous HMPO_2_	Start cold	1	0	0.5	3
	End cold	0.5	0	1	2
	End NR	2	3	3	1
End-ischemic HMPO_2_	Start cold	0.5	0	0	3
	End cold	0	0	2	1.5
	End NR	3	2	2	1
SCS	Start cold	0	0	1	2
	End cold	0.5	0	1	2
	End NR	3	3	3	0.5

Scores of hematoxylin and eosin–stained biopsies were taken at baseline, end of hypothermic preservation, and end of normothermic reperfusion. Each pancreas was scored 0 (none), 1, 2, or 3 (maximum) for necrosis, normal architecture, presence of RBCs in capillaries and arterioles/venules. The scores are illustrated as medians of each group at each timepoint.

HMPO_2_, oxygenated hypothermic machine perfusion; NR, normothermic reperfusion; RBC, red blood cell; SCS, static cold storage.

Immunolabeling for insulin demonstrated the presence of large islets and many smaller clusters of beta cells in all groups (Figure 5D–F). Simultaneous labeling of insulin, glucagon, and somatostatin using fluorescence microscopy (Figure 5G) demonstrated preservation of all islet-cell hormone content with no cross-reactivity.

### Functional Assessment

**FIGURE 6.**
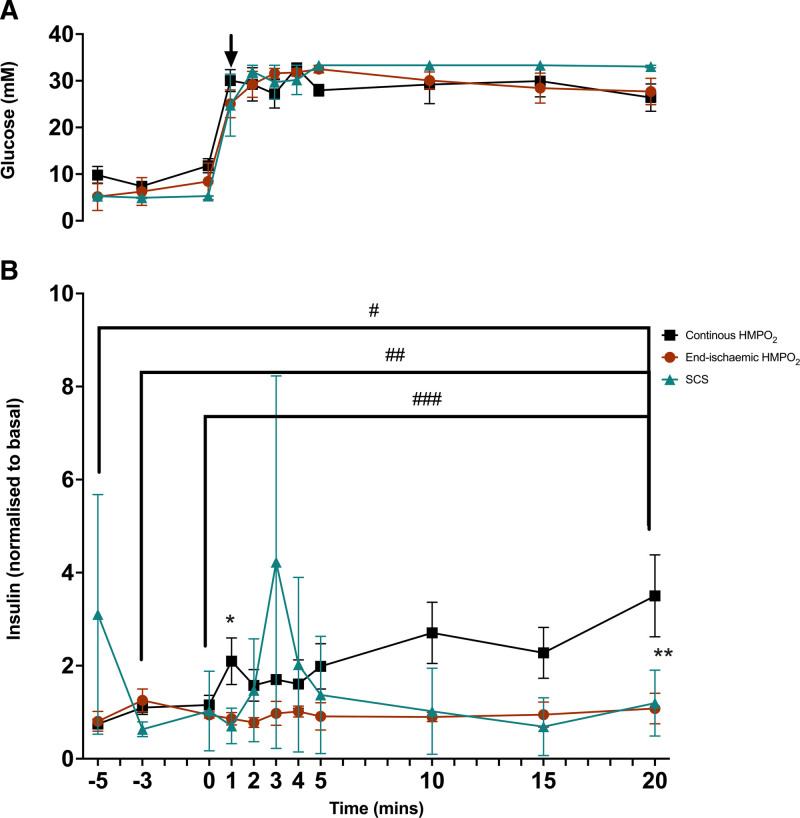
Glucose measurements and glucose-stimulated insulin secretion during normothermic reperfusion after continuous HMPO_2_ (n = 3), end-ischemic HMPO_2_ (n = 3), and SCS (n = 3): The black arrow signifies the administration of glucose. A, Glucose levels in portal venous samples are increased and sustained in all groups from minute 1 onward. B, Insulin secretion (contemporaneous) in all groups, which is significantly higher in the continuous HMPO_2_ group, compared with the end-ischemic HMPO_2_ group (2-way ANOVA with post hoc Tukey test [*P* < 0.02]; difference at minute 1 [*P = 0.02] and at minute 20 [**P < 0.001]). Insulin secretion is shown in mU/L as mean ± SEM. In the continuous HMPO_2_ group, the baseline samples were significantly different from the end of the test: ^**#**^minute –5 to minute 20 (*P* < 0.001), ^**##**^minute –3 to minute 20 (*P* = 0.007), and ^**###**^minute 0 to minute 20 (*P* = 0.0011). Cont, continuous HMPO_2_ group. End, end-ischemic HMPO_2_ group. HMPO_2_, oxygenated hypothermic machine perfusion; SCS, static cold storage

Portal venous effluent samples showed a similarly sharp and sustained rise in glucose levels across all pancreases, ascertaining the comparability of all experiments (Figure 6A). The contemporaneous insulin levels in the effluent demonstrated typical pancreatic islet insulin secretion as a biphasic pattern only in the continuous HMPO_2_ group and were significantly higher than in the end-ischemic HMPO_2_ group (2-way ANOVA, Tukey’s test post hoc, *P* < 0.02; Figure 6B). Insulin secretion was highly variable and not synchronized to glucose levels in either the SCS or end-ischemic group (Figure 6B). The initial phase refers to the peak 1 min after glucose administration, and the second phase, sustained insulin secretion, lasted from 5 to 20 min in response to sustained glucose stimulation. Overall, continuous HMPO_2_ promoted significantly higher cumulative insulin secretion than end-ischemic HMPO_2_ in the initial phase (unpaired *t* test, *P* = 0.01).

## DISCUSSION

This study suggests that continuous HMPO_2_ is a superior method for preserving beta-cell function compared with SCS and end-ischemic HMPO_2_ in a porcine model of circulatory death, assessed by NR with whole blood as a surrogate for transplantation.

### Perfusion and Assessment Models

The porcine abattoir model for solid organ normothermic perfusion has been successfully used by our group to establish protocols for translational clinical trials in kidney^22^ and liver^23^ perfusion studies. Assessment of the reconditioning potential of HMPO_2_ on porcine pancreases using this normothermic perfusion model was chosen because it results in consistent and sufficient organ injury to elicit significant differences. NR is a technique that uses machine perfusion under normothermic conditions using whole blood as a surrogate for transplantation. This allows assessment of the organ morphology and function ex vivo.^14,18,24^

### HMPO_2_ and NR Model Development and Feasibility

HMPO_2_ is a superior modality for other solid organ preservation such as heart, kidney, and liver compared with SCS and has shown promise in multiple preclinical models for pancreas preservation. Previous work on HMPO_2_ of human pancreases resulted in homogeneous perfusion, confirmed by Acridin Orange staining, and an increase in ATP concentration, supporting the hypothesis that oxygen delivery restores cellular energy stores under hypothermic conditions. Tissue analysis showed no evidence of cellular injury, edema, or increased reactive oxygen species after 6 h of perfusion. In that study, 2 pancreases underwent islet isolation and in vitro assessment to confirm islet viability and function.^11^ Allotransplantation of pancreases in a diabetic porcine model after 6 h of hypothermic machine perfusion (HMP) after minimal WIT was successful, demonstrating no vascular damage or thrombosis postmortem.^15^

### HMPO_2_ Durations are Clinically Relevant

The 2 durations of HMPO_2_ were selected on the basis of evidence from clinical trials in liver and kidney transplantation. Two-hour end-ischemic HMPO_2_ has shown improvement in biliary outcomes in liver preservation,^8,25-27^ and continuous HMPO_2_ has shown a lower incidence of rejection and better 1-y graft function in kidney grafts.^9^ In addition, preclinical work published by our group showed that 2-h end-ischemic HMPO_2_ was beneficial over SCS in pancreas preservation.^14^

The 2 HMPO_2_ interventions (n = 12) of continuous and end-ischemic preservation, as well as all NR assessments (n = 18), were feasible with the described protocol.

### Validity and Advantages of Applied Perfusion Models

The 3 groups had comparable WIT and cold ischemic time (CIT), which closely translates to that seen in the clinical setting. The continuous HMPO_2_ group had a longer duration of HMPO_2_, which resulted in an increase in perfusate flow compared with the shorter HMPO_2_ duration in the end-ischemic group; however, during NR, there were no significant differences in perfusion parameters. The presence of RBCs in the capillaries in all the biopsies (n = 18) taken at the end of NR, but not at the start of hypothermic preservation, was evidence of capillary-level perfusion. This affirms the validity of our reperfusion model.

The ethical and financial advantages of using abattoir organs come with variation in the degree of tissue injury sustained during the retrieval process, which is inevitable in this setting but may also reflect biological variability. This model also uses young, healthy animals without cerebral injury, immunosuppression, or alloantigen exposure, which makes it an excellent preclinical option but limits direct translatability.

### Perfusate Biomarkers

Amylase, lipase, and LDH increased over time in all pancreases, but no statistically significant difference was noted between the groups, which is consistent with results seen in previous porcine and nonhuman primate pancreas preclinical HMP and HMPO_2_ studies.^14-16^ Interestingly, the increase of these markers slowed during NR after continuous HMPO_2_.

cfDNA has been shown to reflect degree of injury during ex vivo preservation of lungs^28^ and we investigated it as a reflection of cell death, necrosis, and apoptosis. Although the levels of circulating cfDNA were significantly different between individual pancreases, there was no significant difference in the mean levels between the 3 groups.

When comparing cfDNA within each group at different timepoints, there was a statistically significant decrease from the start to the end of cold preservation in the continuous HMPO_2_ group, which is the only marker of damage during hypothermia to correlate with functional assessment.

In the SCS group, we observed a significant decrease between the start of cold preservation and the end of NR, not cold preservation. As the results were normalized to the baseline value (at the start of cold preservation) for each pancreas, a high starting level in the SCS group may explain this result.

### Lack of Correlation Between Biomarkers and Functional Assessment

The lack of correlation between the measured biomarkers and endocrine function could be explained by the physiological differential microvascular vulnerability between exocrine and endocrine pancreatic tissue, by the distinctly different pharmacokinetics of each biomarker, or by the challenge of interpreting their levels in an ex vivo isolated organ perfusion circuit.

Our amylase, lipase, and LDH results are in line with previously published work in pancreas normothermic perfusion models.^14,29,30^ Pancreas normothermic perfusion, particularly an ex vivo reperfusion model, is expected to result in significant inflammation as a result of reflux excretion and reperfusion injury, leading to exocrine tissue damage. The GSIS here assessed islet function, which is not expected to coincide with early acute exocrine damage.^31^ This lack of correlation highlights the lack of specificity and poor correlation between the enzymatic biomarkers, which are largely used in clinical transplantation, and endocrine function. This further emphasizes the dissociation between exocrine inflammation and endocrine function, as well as the lack of clinically useful biomarkers.

### Undetermined cfDNA Kinetics in an Ex Vivo Circuit

The significant decrease and low absolute values of cfDNA at the end of NR in the SCS group are challenging to explain, but 1 theory is that it reflects significant irreversible reperfusion injury resulting in perfusion shutdown at the microvascular or lobular level, compounded by the short half-life of this marker. This can be as short as a few minutes, potentially shortened further in an ex vivo circuit at 37 °C with whole blood as perfusate.^28,32,33^ This theory is supported by limited evidence available of cfDNA time-dependent changes during ex vivo kidney hypothermic perfusion^34^ and liver and lung normothermic perfusion.^28,35-37^

### Histological Assessment

Histologically, all pancreases showed mild damage at the start of hypothermic preservation, specifically minimal loss of lobular architecture. This could be explained by the warm ischemic injury sustained during retrieval, suggesting that, at baseline, this model was more injured than other animal and human models previously used to investigate HMP or HMPO_2_.^12,13,15-17^ There was no difference demonstrated at the end of hypothermic preservation across the 3 groups; in contrast, there was notably more severe histological evidence of damage at the end of NR, particularly in the SCS group.

### Functional Assessment Differences Between Groups

Despite the small sample size and technical challenges, a clear difference in insulin secretion could still be demonstrated. The continuous HMPO_2_ group exhibited a preserved biphasic insulin secretory response during GSIS, which was not observed in the other 2 groups, suggesting that continuous HMPO_2_ preserved islet function more efficiently. The highly variable insulin secretion in the SCS group can be explained by beta-cell injury. Absolute insulin values were within the range reported for large-animal ex vivo pancreas models and should be interpreted relative to baseline within each graft.^14,17^

Previous work using the same abattoir model showed that end-ischemic HMPO_2_ for 2 h after 3 h of SCS yielded superior perfusion characteristics, lower lactate levels, and improved insulin secretion during GSIS compared with the SCS group.^14^ However, the current study had 5 h of additional CIT, which can explain the difference between our findings.

Furthermore, in a similar study in which porcine pancreases were exposed to 5 h of HMP followed by up to 7 h of SCS, it was shown that 2 pancreases in the HMP group exhibited an insulin response to glucose stimulation, in contrast to none in the SCS-only group (n = 3).^17^

These findings highlight the sensitivity of the porcine pancreas to ischemic injury and how subtle increases in ischemic time can affect beta-cell function. This correlates with the sensitivity of human pancreases to CIT, which is a key factor in posttransplant outcomes.^38^ It also highlights the importance of demonstrating functional difference in preclinical models to support the translation of machine preservation technology into clinical practice.

### Limitations

The primary limitation of this study is the relatively small sample size (n = 18, 6 in each group). This may affect the statistical reliability of the results in particular, markers of damage and histological assessment. Any porcine model has the limitation of subtle differences in pancreatic anatomy and physiology compared with a human model, which therefore affects direct translation. A further limitation is the short duration of the NR model; this may not allow for the full extent of damage to be demonstrated, but our model was sufficient to illustrate a functional assessment, which was the primary aim.

## CONCLUSIONS

Further progress is needed to improve pancreas graft preservation and assessment, increase donor organ utilization, and decrease morbidity associated with pancreas transplantation. Although HMPO_2_ is now suitable for evaluating safety and feasibility in a clinical trial, the best mode of application of this technology is still under investigation.

Continuous HMPO_2_ was a superior method of preservation of the injured pancreases within the limitations of our model, but this comes with the challenge of initiating perfusion at retrieval and the complexity of pancreas surgical preparation required for perfusion at the donation site, which would pose an immense logistical challenge and increase the risk to the graft during transportation.

The practical implementation of end-ischemic HMPO_2_, immediately before transplant, is clearly more feasible, but its functional benefit might depend on the duration of cold ischemia before the start of perfusion. Technological advances—pancreas-specific devices and consumables, improved perfusate composition, integrated hemofiltration systems, and novel therapeutics—together with further consensus on perfusion protocols will increase feasibility and ease the integration of HMPO_2_ into clinical practice.

When HMPO_2_ is successfully applied in clinical pancreas preservation, it has the potential to mitigate the effects of IRI, thereby decreasing the intensity and frequency of posttransplant pancreatitis, decreasing the risk of graft vascular thrombosis, and improving pancreas graft survival and long-term function. Safety and feasibility testing will be an appropriate first step in translating this technology for pancreas preservation into clinical practice.

## References

[1] National Health Service (NHS) England. National Diabetes Audit, 2019-20, type 1 diabetes. NHS Digital. Available at https://digital.nhs.uk/data-and-information/publications/statistical/national-diabetes-audit/national-diabetes-audit-2019-20-type-1-diabetes. Accessed April 10, 2024.

[2] The Diabetes Control and Complications Trial Research Group. The effect of intensive treatment of diabetes on the development and progression of long-term complications in insulin-dependent diabetes mellitus. N Engl J Med. 1993;329:977–986. 8366922 10.1056/NEJM199309303291401

[3] Stratta RJ Gray D Friend P Colman A. Pancreas, Islet and Stem Cell Transplantation for Diabetes. Oxford University Press; 2010.

[4] Parsons J Counter C. Annual report on pancreas and islet transplantation. NHS Blood and Transplant. Available at https://nhsbtdbe.blob.core.windows.net/umbraco-assets-corp/30883/nhsbt-pancreas-and-islet-transplantation-report-2223.pdf. Accessed July 31, 2025.

[5] Page M Rimmelé T Ber CE. Early relaparotomy after simultaneous pancreas-kidney transplantation. Transplantation. 2012;94:159–164. 22728293 10.1097/TP.0b013e318254dae1

[6] Banga N Hadjianastassiou VG Mamode N. Outcome of surgical complications following simultaneous pancreas-kidney transplantation. Nephrol Dial Transplant. 2012;27:1658–1663. 21903603 10.1093/ndt/gfr502

[7] Chouchani ET Pell VR Gaude E. Ischaemic accumulation of succinate controls reperfusion injury through mitochondrial ROS. Nature. 2014;515:431–435. 25383517 10.1038/nature13909 PMC4255242

[8] van Rijn R Schurink IJ de Vries Y; DHOPE-DCD Trial Investigators. Hypothermic machine perfusion in liver transplantation—a randomized trial. N Engl J Med. 2021;384:1391–1401. 33626248 10.1056/NEJMoa2031532

[9] Jochmans I Brat A Davies L; COMPARE Trial Collaboration and Consortium for Organ Preservation in Europe (COPE). Oxygenated versus standard cold perfusion preservation in kidney transplantation (COMPARE): a randomised, double-blind, paired, phase 3 trial. Lancet. 2020;396:1653–1662. 33220737 10.1016/S0140-6736(20)32411-9

[10] National Institute for Health and Care Research. Oxygenated hypothermic machine perfusion in pancreas preservation for transplantation. Available at https://www.fundingawards.nihr.ac.uk/award/NIHR204643. Accessed February 14, 2025.

[11] Leemkuil M Lier G Engelse MA. Hypothermic oxygenated machine perfusion of the human donor pancreas. Transplant Direct. 2018;4:e388. 30498765 10.1097/TXD.0000000000000829 PMC6233671

[12] Branchereau J Renaudin K Kervella D. Hypothermic pulsatile perfusion of human pancreas: preliminary technical feasibility study based on histology. Cryobiology. 2018;85:56–62. 30292812 10.1016/j.cryobiol.2018.10.002

[13] Doppenberg JB Leemkuil M Engelse MA. Hypothermic oxygenated machine perfusion of the human pancreas for clinical islet isolation: a prospective feasibility study. Transpl Int. 2021;34:1397–1407. 34036616 10.1111/tri.13927 PMC8456912

[14] Ogbemudia AE Hakim G Dengu F. Development of *ex situ* normothermic reperfusion as an innovative method to assess pancreases after preservation. Transpl Int. 2021;34:1630–1642. 34448276 10.1111/tri.13990

[15] Prudhomme T Kervella D Ogbemudia AE. Successful pancreas allotransplantations after hypothermic machine perfusion in a novel diabetic porcine model: a controlled study. Transpl Int. 2021;34:353–364. 33275807 10.1111/tri.13797

[16] Prudhomme T Renaudin K Lo Faro ML. Ex situ hypothermic perfusion of nonhuman primate pancreas: a feasibility study. Artif Organs. 2020;44:736–743. 31995645 10.1111/aor.13655

[17] Hamaoui K Gowers S Sandhu B. Development of pancreatic machine perfusion: translational steps from porcine to human models. J Surg Res. 2018;223:263–274. 29325720 10.1016/j.jss.2017.11.052

[18] Moers C van Gelder F Napieralski BP. Machine perfusion or cold storage in deceased-donor kidney transplantation. N Engl J Med. 2009;13:7–19. 10.1056/NEJMoa0802289 19118301

[19] Dengu F Neri F Ogbemudia E. Abdominal multiorgan procurement from slaughterhouse pigs: a bespoke model in organ donation after circulatory death for ex vivo organ perfusion compliant with the 3 Rs (reduction, replacement & refinement). Ann Transl Med. 2022;10:1–1. 35242846 10.21037/atm-21-2494 PMC8825551

[20] Brereton MF Rohm M Shimomura K. Hyperglycaemia induces metabolic dysfunction and glycogen accumulation in pancreatic β-cells. Nat Commun. 2016;7:13496. 27882918 10.1038/ncomms13496 PMC5123088

[21] Thomsen SK Raimondo A Hastoy B. Type 2 diabetes risk alleles in PAM impact insulin release from human pancreatic beta cells. Nat Genet. 2018;50:1122–1131. 30054598 10.1038/s41588-018-0173-1 PMC6237273

[22] Dumbill R Knight S Hunter J. Prolonged normothermic perfusion of the kidney prior to transplantation: a historically controlled, phase 1 cohort study. Nat Commun. 2025;16:4584. 40382321 10.1038/s41467-025-59829-5 PMC12085653

[23] ISRCTN. ISRCTN14957538: normothermic (normal body temperature) machine perfusion to remove fat from donor livers prior to transplantation. doi:10.1186/ISRCTN14957538.

[24] Bral M Dajani K Leon Izquierdo D. A back-to-base experience of human normothermic ex situ liver perfusion: does the chill kill? Liver Transpl. 2019;25:848–858. 30938039 10.1002/lt.25464

[25] Dutkowski P Furrer K Tian Y. Novel short-term hypothermic oxygenated perfusion (HOPE) system prevents injury in rat liver graft from non-heart beating donor. Ann Surg. 2006;244:968–976; discussion 976. 17122622 10.1097/01.sla.0000247056.85590.6b PMC1856639

[26] de Rougemont O Breitenstein S Leskosek B. One hour hypothermic oxygenated perfusion (HOPE) protects nonviable liver allografts donated after cardiac death. Ann Surg. 2009;250:674–683. 19806056 10.1097/SLA.0b013e3181bcb1ee

[27] van Rijn R Karimian N Matton APM. Dual hypothermic oxygenated machine perfusion in liver transplants donated after circulatory death. Br J Surg. 2017;104:907–917. 28394402 10.1002/bjs.10515 PMC5484999

[28] Kanou T Nakahira K Choi AM. Cell-free DNA in human ex vivo lung perfusate as a potential biomarker to predict the risk of primary graft dysfunction in lung transplantation. J Thorac Cardiovasc Surg. 2021;162:490–499.e2. 32928548 10.1016/j.jtcvs.2020.08.008

[29] Ray S Parmentier C Kawamura M. Reanimating pancreatic grafts subjected to prolonged cold ischemic injury using normothermic ex vivo perfusion. Transplant Direct. 2024;10:e1620. 38617463 10.1097/TXD.0000000000001620 PMC11013695

[30] Parmentier C Ray S Mazilescu LI. Normothermic ex vivo machine perfusion of discarded human pancreas allografts: a feasibility study. Transplant Int. 2023;36:10936. 10.3389/ti.2023.10936 PMC10210159 37252614

[31] Śliwińska-Mossoń M Bil-Lula I Marek G. The cause and effect relationship of diabetes after acute pancreatitis. Biomedicines. 2023;11:667. 36979645 10.3390/biomedicines11030667 PMC10044911

[32] Rumore P Muralidhar B Lin M. Haemodialysis as a model for studying endogenous plasma DNA: oligonucleosome-like structure and clearance. Clin Exp Immunol. 1992;90:56–62. 1395101 10.1111/j.1365-2249.1992.tb05831.x PMC1554541

[33] Yao W Mei C Nan X. Evaluation and comparison of in vitro degradation kinetics of DNA in serum, urine and saliva: a qualitative study. Gene. 2016;590:142–148. 27317895 10.1016/j.gene.2016.06.033

[34] Duarte S Fassler AM Willman M. Soluble DNA concentration in the perfusate is a predictor of posttransplant renal function in hypothermic machine perfused kidney allografts. Transplant Direct. 2025;11:e1768. 40124244 10.1097/TXD.0000000000001768 PMC11927653

[35] Yamamoto H Wilson GW Sundby A. Cell-free DNA in ex-vivo lung perfusate is associated with low-quality lungs and lung transplant outcome. J Heart Lung Transplant. 2025;44:1438–1448. 40049261 10.1016/j.healun.2025.02.1693

[36] Stone JP Ball AL Critchley WR. *Ex vivo* normothermic perfusion induces donor-derived leukocyte mobilization and removal prior to renal transplantation. Kidney Int Rep. 2016;1:230–239. 29142927 10.1016/j.ekir.2016.07.009 PMC5678860

[37] Westhaver LP Nersesian S Arseneau RJ. Mitochondrial DNA levels in perfusate and bile during ex vivo normothermic machine correspond with donor liver quality. Heliyon. 2024;10:e27122. 38463874 10.1016/j.heliyon.2024.e27122 PMC10920371

[38] Rudolph EN Dunn TB Sutherland DER. Optimizing outcomes in pancreas transplantation: impact of organ preservation time. Clin Transplant. 2017;31:e13035. 10.1111/ctr.13035 28636074

